# Disentangling the Pathogenesis of Systemic Lupus Erythematosus: Close Ties between Immunological, Genetic and Environmental Factors

**DOI:** 10.3390/medicina59061033

**Published:** 2023-05-26

**Authors:** Henry Sutanto, Yuliasih Yuliasih

**Affiliations:** 1 Department of Internal Medicine, Faculty of Medicine, Universitas Airlangga, Surabaya 60132, Indonesia; henry1988md@gmail.com; 2 Department of Internal Medicine, Dr. Soetomo General Academic Hospital, Surabaya 60286, Indonesia; 3 Division of Rheumatology, Department of Internal Medicine, Faculty of Medicine, Universitas Airlangga, Surabaya 60132, Indonesia

**Keywords:** systemic lupus erythematosus, autoimmune, immune, genetics, environment, rheumatic disease, internal medicine

## Abstract

Systemic Lupus Erythematosus (SLE) is a systemic autoimmune disease that attacks various organ systems with a variety of clinical implications, ranging from mild skin and mucosal manifestations to severe central nervous system manifestations and death. Cases of SLE have been documented nearly two centuries ago when scholars used the terms ‘erythema centrifugum’ and ‘seborrhea congestiva’ to describe the discoid skin lesions and the butterfly or malar rash in SLE. Since then, knowledge about this disease has developed rapidly, especially knowledge related to the underlying pathogenesis of SLE. To date, it is known that immune system dysregulation, supported by genetic and environmental predisposition, can trigger the occurrence of SLE in a group of susceptible individuals. Various inflammatory mediators, cytokines and chemokines, as well as intra- and intercellular signaling pathways, are involved in the pathogenesis of SLE. In this review, we will discuss the molecular and cellular aspects of SLE pathogenesis, with a focus on how the immune system, genetics and the environment interact and trigger the various clinical manifestations of SLE.

## 1. Introduction

Systemic Lupus Erythematosus (SLE) is a systemic autoimmune disease that attacks various organs and exhibits a wide spectrum of clinical manifestations, ranging from mild skin and mucosal manifestations to severe central nervous system manifestations and death [1]. This disease is one of the most common autoimmune diseases found in general practice. The global incidence of SLE is estimated at 5.14 per 100,000 person-years, with a varying incidence in various studies from 1.4 to 15.13 per 100,000 person-years. Meanwhile, at the regional level, the incidence of SLE in the general population varies from 1.18 per 100,000 person-years in Central Asia to 13.74 per 100,000 person-years in Central Europe [2]. It predominantly affects females with a 9:1 female-to-male incidence ratio, especially during peak reproductive years (20–30 years of age) [3]. While it is more frequent in females, SLE has been shown to have more severe clinical manifestations in males than in females [4]. 

In 2014, the age-standardized death rate from SLE was reported at around 2.7 deaths per 1 million population: 4.5 deaths per 1 million population in the female group and 0.8 deaths per 1 million population in the male group. These mortality rates vary widely, with several hundredfold heterogeneity between countries, with the highest age-standardized mortality rates being in Latin America and the lowest in Europe. Our own data at Dr. Soetomo General Academic Hospital Surabaya showed that around 30% of SLE patients in the inpatient ward suffered from severe SLE, with a mortality rate of 22.9% [5], which was considerably higher than previously reported disease survival [6]. This variation between countries or regions can be due to reporting bias, differences in disease severity, socioeconomic factors, and treatment capacity in each country or region [7,8]. The most common cause of death is infectious diseases, followed by cardiovascular diseases, and interestingly, both high and low-income countries reported infectious diseases as the highest cause of death in SLE patients [7,9]. 

SLE cases have actually been documented nearly two centuries ago by Biett, Cazenave and Hebra, where they used the terms ‘erythema centrifugum’ and ‘seborrhea congestiva’ to describe discoid skin lesions and the butterfly or malar rash in SLE [10]. Since then, knowledge about this disease has developed rapidly, especially knowledge related to the underlying pathogenesis of SLE. Up to this point, it is known that immune system dysregulation, supported by genetic and environmental predisposition, can trigger the occurrence of SLE in a group of susceptible individuals. Various inflammatory mediators, cytokines and chemokines, as well as intra- and intercellular signaling pathways, have been reported to be involved in the pathogenesis of SLE. In this review, we aim to discuss the molecular and cellular aspects of SLE, with a focus on the role of the immune system, genetics and the environment. Concurrently, we sought to better understand how the complex interactions at the molecular and cellular levels can trigger various clinical manifestations of SLE.

## 2. Apoptosis and Immune System Dysregulation Are Hallmarks of SLE Pathogenesis

### 2.1. Dysregulation of Innate Immune Response

**Figure 1:**
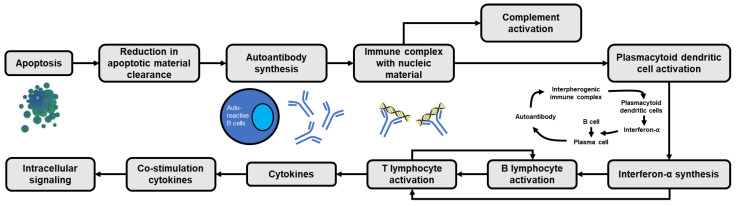
Cascade of apoptosis-induced immune activation in SLE. The vicious cycle of interferon-α generation is present as a result of the continuous transformation of B cells into autoantibody-forming plasma cells.

Under physiological conditions, adequate removal of apoptotic debris can prevent self-antigen exposure and activation of immune cells. However, increased apoptotic rates or low clearance of apoptotic debris in SLE (e.g., impaired phagocytosis due to dysfunction of immune tolerance) will lead to increased formation of autoantigen-antibody complexes by autoreactive B cells (Figure 1) [11]. Initially, the innate immune system (e.g., macrophages, granulocytes and natural killer [NK] cells) via pattern recognition receptors (PRR; e.g., toll-like receptor [TLR], nucleotide binding and oligomerization domain receptor [NLR] and retinoid acid-inducible gene [RIG]-I-like receptor [RLR]) will recognize apoptotic bodies or damaged cell particles [12]. Ligand binding with TLRs can activate B lymphocytes and generate autoantibodies which will form immune complexes (antigen-antibody complexes) which can then be recognized by plasmacytoid dendritic cells (pDC) via their surface receptor, FcγIIa. The immune complexes that are formed can simultaneously activate the complement system. Then, pDCs induce the production of endogenous type I interferon (IFN) (e.g., IFN-α) via a TLR7 and TLR9-dependent pathway. In SLE patients, TLR9 ligands can activate pDCs via the interferon regulatory factor (IRF) signaling pathway, generating large amounts of type I IFNs. Additionally, activation of pDC by TLR7 ligand will increase the expression of interleukin (IL)-1β and IL-23, and stimulate Th17 differentiation [13,14].

IFN-α that is formed can activate B cells (e.g., by affecting the differentiation of B cells into antibody-generating plasma cells) and T cells (Figure 1), as well as affect the maturation of DCs (i.e., the transformation of monocytes into DCs) and NK cells. Furthermore, activated DCs will recognize the antigen and produce IFN-α, further increasing circulating IFN-α levels (Figure 1) [15,16,17,18]. In addition, IFN-α can activate T helper (Th) cells, increasing DC antigen presentation ability, which in turn induces the production of cytokines such as IL-1, IL-2, IL-4, IL-6 and IL-8 [17]. Meanwhile, the activation of the NLR will activate the production of other pro-inflammatory molecules, such as the inflammasomes and caspases, which will produce IL-1β and IL-18 [17,19].

Neutrophils also have a significant role in the pathogenesis of SLE. Neutrophil activation will induce the release of proteases, tissue damage factors and reactive oxygen species (ROS) which are the causes of tissue damage in SLE. Activated neutrophils also release large amounts of cytokines and chemokines, which can interfere with the regulation of the immune system. In addition, active neutrophils will release neutrophil extracellular traps (NETs) which are fibrous woven composed of core and granular components of activated neutrophil cell membranes which function to trap, inhibit and kill extracellular pathogens. NETs also contain chromatin, histones, granules, enzymes (e.g., myeloperoxidase [MPO], neutrophil elastase [NE], cathelicidins such as LL-37, peptidyl arginine deiminase 4 [PAD4], matrix metalloproteinase 9 [MMP-9], proteinase 3, cathepsin, lactoferrin, and gelatinase), calprotectin and lysosomal membrane protein-2. NETs are important components in the pathogenesis of SLE. A large number of NETs are found in the kidney, skin and blood of SLE patients, and their presence correlates with disease activity. In SLE, suboptimal clearance and/or excessive formation of NETs (NETosis) due to soluble mediators or microvesicles released upon endothelial or platelet activation will result in autoantigen externalization and induce type I IFN synthesis and endothelial damage [17,20,21].

In addition, increased peptidyl arginine deiminase 4 (PAD4) in SLE stimulates histone citrullination which stimulates the formation of autoantigens and deamination of proteins such as antithrombin, histone H2A, histone H3 and histone H4 [21]. Histone components in NETs are able to stimulate the activation of the innate immune system by activating TLRs and the NLRP3 inflammasome. NETs also activate caspase-1 which causes active release of IL-1β and IL-18, and then joins complement C1q to activate the classical complement pathway. Meanwhile, activated complement can inhibit the degradation of NETs and even exacerbate autoimmunity [17]. In addition, NETs can also induce an adaptive immune system through activation of pDC via TLR7 and TLR9, and play a role in priming T lymphocyte cells by lowering their activation threshold, making T cells sensitive to specific antigens, even at suboptimal exposure [21].

### 2.2. Dysregulation of Adaptive Immune Response

**Figure 2:**
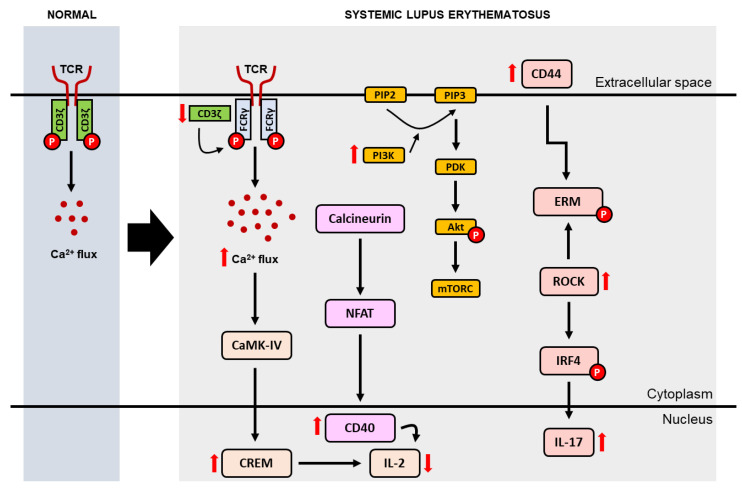
Modifications of T cell receptor complex leading to a reduction in IL-2 and an increase in IL-17 in SLE. (CaMK-IV = calcium/calmodulin-dependent protein kinase type IV; CD = cluster differentiation; CREM = cyclic adenosine monophosphate responsive element modulator; ERM = ezrin/radixin/moesin; IL = interleukin; IRF4 = interferon regulatory factor 4; mTORC = mammalian target of rapamycin complex; NFAT = nuclear factor of activated T cells; PDK = phosphatidylinositide-dependent protein kinase; PI3K = phosphoinositide 3-kinases; PIP2 = phosphatidylinositol 4,5-bisphosphate; PIP3 = phosphatidylinositol 3-Phosphate; ROCK = rho-associated protein kinase; TCR = T cell receptor).

Impaired immune tolerance (i.e., the state of the immune system that is active but not responsive to self-antigens or to certain antigens that can induce an immune response in the body [22]) plays an important role in the pathogenesis of SLE. In addition to causing abnormalities in cytokine secretion and intracellular signal transduction, impaired immune tolerance can also cause impaired recruitment and activation of B lymphocyte cells and dendritic cells [17,20]. T lymphocyte cells, for example, play an important role in the pathogenesis of SLE by inducing inflammation through the secretion of pro-inflammatory cytokines, inducing B cells to produce autoantibodies and maintaining the existence of the disease through the formation of a collection of memory T cells that are autoreactive [15]. In SLE, autoreactive T cells can increase the expression of apoptotic ligands such as tumor necrosis factor-related apoptosis-inducing ligand (TRAIL), tumor necrosis factor-like weak inducer of apoptosis (TWEAK) and Fas ligand (FasL) which will directly facilitate monocyte apoptosis [20,23]. Meanwhile, expression of cluster differentiation (CD) 3ζ (i.e., a molecule in the T cell receptor [TCR] complex and CD3 that plays a role in regulating the influx of calcium into T cells) is significantly decreased in SLE (Figure 2), causing a rearrangement of the TCR-CD3 complex, where CD3ζ is substituted by the homologous Fc receptor common gamma subunit chain (FcRγ). FcRγ recruits splenic tyrosine kinase, fostering the influx of calcium into T lymphocytes. Elevated intracellular calcium causes an increase in calcineurin activation which in turn dephosphorylates cytoplasmic nuclear factor of activated T cells (NFAT). Subsequently, dephosphorylated NFAT will translocate to the nucleus to activate gene promoters CD40L and T cells. Meanwhile, activated calmodulin kinase IV (CaMK-IV) increases intranuclear expression of cAMP-responsive element modulator (CREM)-α and inhibited IL-2 production [17].

In addition to reduced expression of CD3ζ, expression of CD44 (i.e., a cell surface molecule involved in T cell activation and adhesion) was found to be increased in SLE and correlated with disease activity. In carrying out its function, CD44 binds to the protein ezrin/radixin/moesin (ERM) which is phosphorylated by rho-associated protein kinase (ROCK). Due to the increase in CD44 in SLE, ERM phosphorylation also increases. Consequently, T cell adhesion and migration are also increased in SLE. In addition, ROCK can also activate IRF4, affect Th17 differentiation, and modulate IL-17 and IL-21 production via the RhoA-ROCK-IRF4 pathway [17,24]. Phosphoinositide-3 kinase (PI3K) activity was also reported to increase in SLE, inducing the phosphatidylinositol-3,4,5-triphosphate (PIP3)/Akt/rapamycin molecular target (mTOR) cascade which in turn stimulates the synthesis of proteins that play a role in division, proliferation and T cell survival [17].

Meanwhile, Th cell dysfunction and imbalance between Th1 and Th2 components, as well as Th17 and Treg have also been implicated in the pathogenesis of SLE. Several studies reported the dominance of Th2 in SLE which causes excessive B cell activation, the formation of autoantibodies and tissue injury [25,26], while other studies documented the opposite condition that Th1 was more dominant in chronic SLE, especially the one manifesting as lupus nephritis [27,28,29]. We also reported a positive relationship between the Th17/Treg ratio and disease activity in SLE [30]. In addition, in SLE there is an increase in Th17 expression, which in turn will increase the levels of IL-17, IL-21, IL-22 and IL-23. IL-17 functions to regulate B cell differentiation and survival, which will then enhance humoral and autoantibody immunity [17,26,31]. High initial serum IL-17 levels can predict poor histopathological outcomes after immunosuppressive therapy in SLE patients [24].

In SLE, there is also the pathological expansion of follicular Th cells which function as inducers of the germinal center, proliferation, isotype exchange and somatic hypermutation, as well as producing IL-21 which plays a role in the differentiation of B cells into memory B cells and antibody-producing plasmablasts. Pathological expansion of follicular Th cells caused by interactions between follicular Th cells and OX40 ligands expressed on antigen-presenting cells will increase antibody production and loss of immune tolerance in SLE patients. Of note, the expression of this OX40 ligand is affected by TLR7 activation [15]. In addition, the number of Treg, which controls self-tolerance and autoimmunity, was reported to decrease in SLE [17].

In addition to T cells, SLE also impairs B cell activity. There is an abnormal tolerance of central and peripheral B lymphocytes in SLE patients. A large number of self-reactive B cells generate SLE-inducing autoantibodies. Several studies have shown that the number of Breg cells (i.e., B lymphocytes that play a role in suppression of the immune response, maintenance of immune tolerance, limitation of active immune responses and restoration of immune system homeostasis [32]) was lower in SLE patients (particularly in patients with lupus nephritis) than in healthy individuals and the number of these cells was restored following treatment with immunosuppressants. Additionally, Breg responses to CD40 stimulation and IL-10 secretion were reduced in the peripheral blood of SLE patients, indicating Breg dysfunction in SLE [17]. Other abnormalities in the B cells of SLE patients include an imbalance in the number of B cell subtypes with an increase in memory B cells relative to naïve B cells, as well as an exaggerated B cell receptor (BCR) response with receptor cross-linking which leads to increased calcium influx and tyrosine phosphorylation of downstream signaling molecules [33]. The two issues interact, in that an increase in the number of memory B cells lowers the threshold for B cell activation, allowing autoreactive B lymphocytes to thrive with minimal antigen contact, while increased BCR activation contributes to a stable active phenotype in SLE [33,34].

In SLE patients, there may also be increased expression of recombination activating genes (RAG) in peripheral B cells which causes BCR mutations and produces autoreactive B cells, as well as shortening of the interaction of T cells and B cells in the germinal center which causes increased autoreactive B cell survival. Other immunological anomalies associated with B cell autoreactivity in SLE include increased plasma cell differentiation and survival, upregulation of TLR signaling, and expression of B cell cytokines such as B cell activating factor (BAFF), IL-6, and IL-21 [15,34]. SLE patients with high BAFF have higher levels of anti-dsDNA, anti-histone, and anticardiolipin antibodies [15].

## 3. The Role of Genetic Predisposition in the Pathogenesis of SLE

### 3.1. Human Leukocyte Antigen (HLA)-Associated Genetic Variations in SLE

Of all the genetic loci related to SLE, the major histocompatibility complex (MHC) region on chromosome 6p21 is the most polymorphic region in the human genome. This region consists of class I (encode HLA-A, -B, -C, -E, -F and -G), class II (encode HLA-DP, -DM, -DO, -DQ and -DR) and class III (encoding complement system and inflammatory genes) MHC genes. Class I MHC molecules are present in all nucleated cells and are responsible for presenting endogenous cytoplasmic antigens, such as viral particles. The antigen is then presented to CD8 T cells which will kill cells infected with the virus. Meanwhile, class II MHC molecules are expressed by antigen-presenting cells (e.g., B cells, macrophages and dendritic cells) and bind to antigens originating from outside the cell (exogenous) through the process of endocytosis and present these antigens to CD4 Th cells [35].

SLE association signals in the MHC region are primarily located in HLA-DRB1 in the MHC class II region or HLA-DRB1-associated long-range HLA gene haplotypes in several ancestral populations [36,37,38]. Genetic variation in these HLA genes is a contributor to the formation of autoantibodies and plays a role in tissue damage due to lupus [39,40,41]. A study conducted on 61 SLE patients in Pakistan who met at least 4 American College of Rheumatology (ACR) criteria stated that there was an increased frequency of several HLA-A, HLA-B and HLA-DRB1 among SLE patients, reflecting a positive association of those alleles with SLE [42]. Similarly, another study with an SLE patient population in Malaysia also demonstrated the involvement of HLA-DRB1 in SLE pathogenesis. SLE carriers with the HLA-DRB1*04 allele experienced increased levels of cytokines (IFNγ, GM-CSF, IL-17F, IL-18, IL-21, and VEGF) and significantly decreased levels of IL-5 and free radicals when compared to carriers SLE without the HLA-DRB1*04 allele [43]. Interestingly, certain alleles of HLA-DRB1 are also determinants of SLE autoantibodies and phenotypes, e.g., HLA-DRB1*03 associates with anti-SSA/Ro60/Ro52/SSB autoantibodies and frequently manifests as discoid lesions in SLE, HLA-DRB1*15 is associated with anti-nucleosome/SmRNP/DNA/RNPA autoantibodies and manifests in the form of lupus nephritis, whereas HLA-DRB1*04 is associated with anti-β2GPI-IgG/anti-CL–IgG/IgM autoantibodies and often occurs in the form of vascular lesions [37]. Another study reported that HLA-DRB1*08 and HLA-DRB1*11 are associated with central nervous system manifestations in SLE, and HLA-DRB1*10 is associated with hematological manifestations in SLE, while HLA-DRB3 is associated with serositis in SLE [44]. The association among HLA, ethnicities and SLE is also evident [45,46]. For example, HLA-DRB1*0301 was commonly found in Caucasians, while DRB1*1503 and DRB1*08 alleles were often discovered in African Americans and Hispanics. Interestingly, the presence of these alleles in certain ethnicities was linked to distinct manifestations of SLE in those populations [45]. In addition, HLA-DR2, HLA-DR3, several non-classical HLA genes, MHC class III genes and single-nucleotide polymorphism (SNP) MHC have also been reported to be associated with SLE [15,38].

### 3.2. The Role of Non-HLA-Associated Genetic Variations in the Pathogenesis of SLE

Another example of a genetic mutation that is rare but carries a high risk of increasing SLE susceptibility is one that inhibits the production of molecules involved in complement activation, including C1q, C2 and C4. Deficiencies in the production of these molecules decrease the ability of the immune system to clear cellular debris, which consequently increases the number of cellular fragments containing nucleic acids. In addition, complement C1q is also responsible for protecting against SLE by directing stimulated immune complexes to monocytes, not to IFN-α-generating pDCs. As a result, inhibition of complement C1q will interfere with the process of directing this immune system activation pathway, and, as a result, pDC will be activated, which will produce IFN-α. Several studies have also reported the association of MHC 8.1, HLA-B8 and HLA-DR3 haplotypes with susceptibility to SLE through modulation of complement C4 [47].

Mutations in genes encoding nuclease enzymes (e.g., TREX1) that cleave DNA or RNA have also been documented in SLE. These mutations and genetic associations support the role of stimulatory cytoplasmic nucleic acids as triggers of immune system activation in SLE. A number of SLE-related SNPs have also been reported, some of which are found in protein-coding genes involved in the induction of type I IFN. In addition, genetic mutations in the IRF5 and IRF7 genes involved in the TLR signaling pathway are deemed responsible for the activation of the innate immune system [47].

Gain-of-function mutations in TLR7, a sensor for viral RNA that functions to bind guanosine, also play a role in the pathogenesis of SLE by increasing the affinity for guanosine nucleosides and cyclic nucleotide cGMP. Such TLR7 mutations also interfere with TLR7 signaling, causing abnormal prolongation in the survival of BCR-expressing B cells, accumulation of CD11c+-expressing B cells in the germinal center of lymphoid organs, as well as an increase in follicular and extrafollicular T helper cells. Of note, a deficiency in MyD88, a protein downstream of TLR7, can prevent these effects [48]. Other SLE-associated gene variants alter the threshold for lymphocyte activation or immune cell signaling efficiency. These SLE-associated variants encode proteins involved in cytokine signaling (e.g., signal transducer and activator of transcription 4 [STAT4]) and in the efficiency of signaling downstream of T- and B cell surface antigen receptors (e.g., protein tyrosine phosphatase non-receptor type 22 [PTPN22], tyrosine-protein kinase LYN, B cell scaffold protein with ankyrin repeats [BANK], B lymphocyte tyrosine kinase [BLK] and tumor necrosis factor-α-induced protein 3 [TNFAIP3]) [15,47]. In addition, SLE-related variants in the kallikrein encoding gene are associated with protection from or susceptibility to kidney damage, and overexpression of Klk1 in the kidney of a mouse model of SLE can reduce SLE-induced inflammation and oxidative damage [47]. 

To date, almost a hundred susceptibility loci have been linked with SLE through genome-wide association studies (GWAS), including the cytotoxic T lymphocyte associated protein-4 (CTLA4) and TNF receptor-associated factor-3 (TRAF3) [49,50,51]. CTLA4 modulates T cell activation, induces immune tolerance, as well as inhibits T cell proliferation dan differentiation, pro-inflammatory cytokines production and cell cycle progression [52]. Meanwhile, TRAF3 modulates the activation of type I IFN and prevents the activation of mitogen-activated protein kinase (MAPK) and nuclear factor kappa B (NF-κB) [53]. 

## 4. The Role of Environmental Triggers in the Pathogenesis of SLE

Exposure to environmental factors, such as ultraviolet B (UV-B) radiation, silica, cigarette smoke, oral contraceptives, infections and toxins, can impair immune tolerance in individuals with genetic susceptibility and cause aberrant autoimmunity activation [15]. The mechanisms underlying this decrease in immune tolerance include increased oxidative stress, upregulation of inflammatory cytokines, systemic inflammation and epigenetic modification, all of which can increase the risk of developing SLE in susceptible individuals.

### 4.1. Pathogenic Microorganism Infection

The genetic material of pathogenic microorganisms acts as a type I IFN stimulator to activate the NF-κB pathway and produce IFN-α which stimulates the immune system to produce autoantibodies. In addition, several mechanisms are known to underlie the involvement of infectious agents in the pathogenesis of SLE including molecular mimicry, epitope spreading, superantigen production, bystander activation, persistent viral infection, disruption of the apoptotic process, clearance deficiency and epigenetic changes (e.g., DNA and microRNA methylation) [54]. Several viruses have been reported to play a role in the pathogenesis of SLE including Epstein-Barr virus (EBV), cytomegalovirus (CMV), parvovirus B19, human immunodeficiency virus-1 (HIV-1) and hepatitis B virus vaccine [15]. Interestingly, several pathogens are reported to have a protective effect on SLE, for example, Plasmodium sp. and Toxoplasma gondii [54,55]. There are at least three mechanisms that could underlie such a protective effect: first, antigenic competition leads to a decreased response to autoantigens; second, suppression of the host immune response to self or non-self molecules by Treg cell subgroups; and third, TLR signaling directly or indirectly regulates the immune suppression of Treg cells [54].

One of the viruses known to influence the risk of SLE is EBV. In EBV infection, deficiencies in the complement pathway, components of the IFN pathway, and MHC-specific alleles may be involved. In addition, a process of molecular mimicry occurs in EBV infection. Antibodies to the EBV nuclear antigen (EBNA-1) can cross-react with SLE-associated autoantigens. In susceptible individuals, the immune response to EBNA-1 leads to the formation of cross-reactive antibodies, followed by epitope dissemination, which eventually develops into SLE [15]. Whereas malaria infection is thought to be able to weaken lupus nephritis in experimental animals by reducing oxidative stress in the kidneys and increasing the antioxidant defense system, while toxoplasma infection can slow the development of autoimmune nephropathy in mice susceptible to lupus by modulating mouse autoantibodies [54]. In addition, repeated use of hydroxychloroquine to treat malaria can delay the onset of SLE in people living in malaria-endemic areas, considering that hydroxychloroquine is one of the drug regimens used in the management of SLE [55,56].

### 4.2. UV-B Radiation Exposure

Exposure to UV-B radiation induces the formation of reactive oxygen species and increases the expression of pro-inflammatory cytokines, such as IFN-α, IL-1, IL-6 and tumor necrosis factor (TNF)-α. In addition, UV-B also increases intercellular adhesion molecules (e.g., ICAM-1 and LAF-1), IL-8 ligand secretion and chemokines (e.g., chemokine (C-C motif) ligand [CCL]-5, CCL20, and CCL22), which recruits immune cells to areas of inflammation [15]. Previous studies reported that there was an upregulation of type I IFN in the skin of SLE patients after exposure to UV light [15,57]. Exposure to UV-B radiation also causes activation of T lymphocyte cells that lasts longer, which is caused by upregulation of type I IFN and repression of Treg cells due to exposure to UV-B light [57]. SLE patients also experience an inhibition of DNA methylation (hypomethylation) and DNA methyltransferase-1 (DNMT1) mRNA expression due to exposure to UV-B light, which causes autoreactivity and production of autoantibodies [15,58].

### 4.3. Oral Contraceptives and Hormonal Therapy

Sex hormones are known to influence immune system function and act as triggers or protectors in the development of SLE. Previous studies have shown an increased risk of SLE associated with estrogen exposure, while progesterone and testosterone play a protective role by counteracting the effects of estrogen [15]. A double-blind, randomized, noninferiority trial concluded that combined oral contraceptives containing low-dose synthetic estrogen and progestin are safe and do not increase the risk of flare in premenopausal women with stable SLE [59]. However, a case-control study reported that combined oral contraceptives are associated with an increased risk of SLE in women who are just starting oral contraceptives [60]. In addition, the influence of sex hormones on disease activity is evident in exacerbations during puberty, pregnancy, and postpartum. Estrogen activity may contribute to the development of SLE by increasing the production of type I IFN, prolonging the survival of autoreactive B cells through the production of anti-dsDNA antibodies, dysregulation of Treg lymphocytes, modulation of TLR pathways, and development of dendritic cells [15].

### 4.4. Silica Dust Exposure

Silica crystals, commonly found in rocks, sand, bricks, concrete and other construction materials, induce cellular apoptosis and intracellular antigen release. In addition, exposure to silica dust also causes an increase in pro-inflammatory cytokine activity, oxidative stress, and reduced Treg cell activity [61]. In animal experiments, exposure to silica was associated with higher levels of serum autoantibodies, antinuclear antibodies (ANA) and immune complexes [15,62].

### 4.5. Smoking and Cigarette Smoke Exposure

Exposure to toxic components of cigarette smoke (e.g., nicotine, polycyclic aromatic hydrocarbons, carbon monoxide and free radicals) can cause oxidative stress and directly damage endogenous proteins and DNA, causing genetic mutations that have the potential to induce autoimmunity and increase the production of pro-inflammatory cytokines [15,63]. Cigarette smoke increases the expression of the membrane receptor Fas (CD95) on the cell surface of B lymphocytes and CD4 T lymphocytes. This CD95 function transmits signals for apoptotic lymphocytes so that with increased expression of CD95 on their surface, B lymphocyte cells are more sensitive to apoptotic signals and more susceptible to undergoing unnecessary apoptosis. This has the potential to induce autoimmunity by overburdening the apoptotic debris clearance mechanism, as mentioned in the previous section. In addition, cigarette smoke has been shown to impair T cell function, reduce NK cells, and impair humoral and cellular immunity. In addition, cigarette smoke can affect T cell function in SLE pathogenesis, including Th17 and Th22 cell function, through aryl hydrocarbon receptors (AhR), which are activated by benzopyrene in cigarette smoke [63]. Additionally, tar and particulate matter from cigarette smoke contain high concentrations of free radicals. Free radicals can interact with DNA and cause epigenetic changes and mutations. Reactive oxygen species produced by the metabolism of tobacco smoke can also damage DNA, resulting in increased production of anti-dsDNA autoantibodies [63].

Several meta-analyses evaluating smoking as a risk factor for SLE found a significant risk for SLE development among smokers compared to nonsmokers [64,65]. A cohort study involving more than 238,000 women reported that the increased risk of SLE mainly only occurred among current or former smokers who had quit smoking within the last 4–5 years [63,64].

### 4.6. Poisons, Drugs and Other Toxic Substances

Drugs previously reported to trigger drug-induced lupus erythematosus (DILE [66]) are hydralazine, procainamide, isoniazid, minocycline, proton pump inhibitors (PPIs) and TNF-α inhibitors [15,67,68]. Some of the potential mechanisms of DILE include genetic predisposition, drug biotransformation and epigenetic changes in immune cells. Procainamide, hydralazine, and isoniazid are mainly metabolized by acetylation using the N-acetyltransferase enzyme. Slow acetylators with genetic N-acetyltransferase deficiency are prone to accumulation of autoantibodies after exposure to these drugs. Several studies have suggested a relationship between HLA-DR2, HLA-DR3, HLA-DR4, C4A and C4B null complement alleles and DILE [15,66]. These drugs can also inhibit complement component C3, which in turn inhibits immune complex clearance.

Meanwhile, biotransformation, another potential mechanism of DILE, is related to the oxidative metabolism of drugs in neutrophils. It is thought that the oxidative metabolism of procainamide and isoniazid releases several toxic metabolites together with myeloperoxidase and reactive oxygen species, which induce cytotoxic effects. Biotransformed drugs and their metabolites have been reported to alter the epigenetic properties of immune cells. Hydralazine and procainamide inhibit T cell DNA methylation, increasing the expression of lymphocyte function-associated antigen 1 (LFA-1), which consequently induces autoreactivity. Autoreactive T cells can then stimulate excessive production of autoantibodies through interactions with MHC class II self molecules on B cells and induce macrophage apoptosis by interacting with class II self MHC molecules on macrophages which release apoptotic chromatin which is highly antigenic from dying macrophages. The innate immune response is also involved in the pathogenesis of DILE. NET is secreted by active neutrophils and contains nuclear DNA and cytosolic proteins. Procainamide and hydralazine stimulate NET formation via the activation of neutrophil muscarinic receptors, inducing an autoimmune response from autoantigen exposure [15,66].

## 5. Conclusions

**Figure 3:**
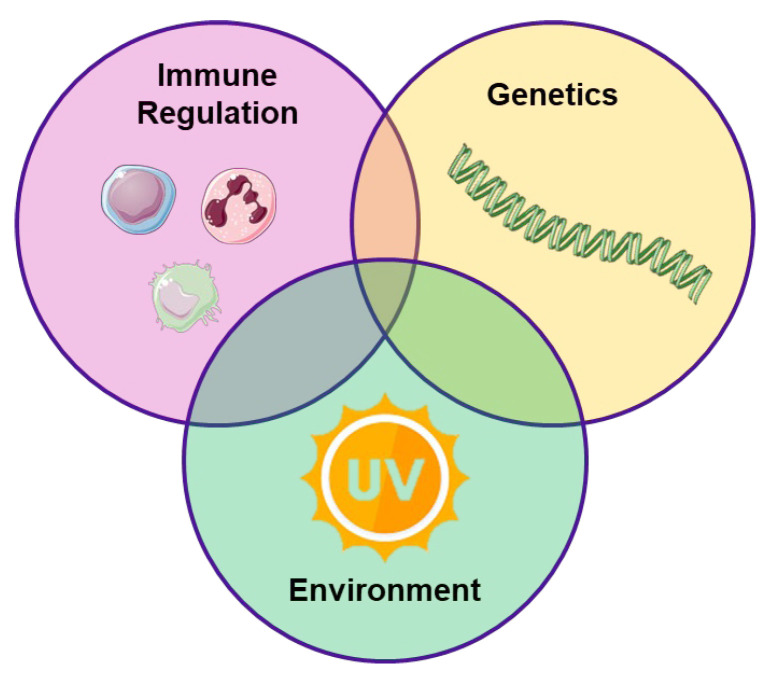
Close ties between immune system, genetics and environment in the pathogenesis of SLE.

SLE is a systemic autoimmune disease that affects various organ systems and has a wide range of clinical manifestations, ranging from asymptomatic to severe and life-threatening consequences. The underlying pathogenesis of SLE is complex and involves many components at the molecular and cellular levels. Dysfunction of the innate (e.g., neutrophils, monocytes, pDCs, NK cells and complement) and adaptive (i.e., B cells and T cells) immune systems, together with genetic predisposition and supported by environmental factors, may precipitate SLE in susceptible individuals (Figure 3). Several genes, both HLA-related and non-HLA-related, are known to play a role in the pathogenesis of SLE. Meanwhile, environmental factors such as UV-B rays, infections, hormonal status and oral contraceptives, exposure to pollutants, and cigarette smoke can increase the risk of SLE. Furthermore, the use of drugs can also induce DILE which has clinical manifestations similar to SLE. Therefore, control of environmental factors accompanied by a stabilization of the immune system, including restoration of immunological tolerance, is important in the prevention and management of SLE in the present and the future.

## Data Availability

No new data were created.
